# Assessing Type 2 Diabetes Risk in the Post-Pandemic Era: A Pharmacy-Led FINDRISC Screening Study

**DOI:** 10.3390/life14121558

**Published:** 2024-11-27

**Authors:** Victoria Bell, Ana Rita Rodrigues, Vera Costa, Catarina Dias, Márcia Alpalhão, Inês Martins, Mário Forrester

**Affiliations:** 1 Social Pharmacy and Public Health Laboratory, Faculty of Pharmacy, University of Coimbra, 3000-548 Coimbra, Portugal; art.rodrigues@ff.uc.pt (A.R.R.); veracosta2001@gmail.com (V.C.); 2 LAQV-REQUIMTE, Group of Pharmaceutical Technology, Faculty of Pharmacy, University of Coimbra, 3000-548 Coimbra, Portugal; 3 Glow—Pharmaceutical Products, 2855-386 Corroios, Portugal; farmaciabentolino@gmail.com (C.D.); marciaalpalhao@gmail.com (M.A.); inespmartins98@gmail.com (I.M.); 4 UFUP—Unidade de Farmacovigilância, Universidade do Porto, 4200-450 Porto, Portugal

**Keywords:** community pharmacy, diabetes, FINDRISC, public health, COVID-19

## Abstract

Diabetes mellitus (DM) is a major global health issue, with type 2 diabetes (T2D) accounting for over 90% of cases. Community pharmacies, given their accessibility, are well positioned to assist in early detection and management of T2D. This study evaluated post-pandemic T2D risk in a Portuguese population using the Finnish Diabetes Risk Score (FINDRISC) across five community pharmacies. A total of 494 participants aged 40 or older without a prior diagnosis of diabetes were assessed. The mean FINDRISC score was 12.3, and 29.8% were identified as high or very high-risk, with 8.7% referred to general practitioners for follow-up based on elevated glycated hemoglobin (HbA1c). Key risk factors include age, body mass index, waist circumference, lack of physical activity, and family history of diabetes. Lower educational levels were also associated with higher diabetes risk. Community pharmacies are shown to play an essential role in screening and educating at-risk populations, emphasizing the importance of physical activity, healthy diets, and regular monitoring. These findings reinforce the value of community pharmacists in mitigating T2D risk and enhancing public health outcomes through cost-effective, validated screening tools like FINDRISC. Finally, pre-pandemic FINDRISC studies discussed show similar results suggesting that the COVID-19 pandemic did not significantly impact the overall risk profile for T2D.

## 1. Introduction

Diabetes mellitus (DM) is one of the most common noncommunicable diseases. Recognized as a global health challenge [1], impacting worldwide an estimated 536.6 million adult patients (20–79 year olds) in 2021 [2], it poses significant health [3] and substantial financial burdens [4] on individuals and society.

According to the latest report from the *Observatório Nacional da Diabetes* (Nacional Diabetes Observatory), in 2021, the estimated prevalence of diabetes in Portugal (20–79 year olds), was 14.1% [5]. Despite increased awareness, the prevalence of the disease in Portugal has risen 20.5% since 2009 and still remains undiagnosed in 6.2% of the population [5].

Classified into four major categories, type 1 (T1D), type 2 (T2D), gestational, and specific types [6], diabetes is a complex chronic metabolic condition in which insulin is not produced or is used inadequately, resulting in elevated blood glucose levels that lead, over time, to micro and macrovascular complications [7].

T2D is commonly asymptomatic and accounts for more than 90% of the diabetes mellitus cases [7,8]. Adults with 40 or more years of age and with a body mass index (BMI) over 30 are at greater risk of developing T2D [9,10,11]. Early detection of the disease can help reduce the severity of associated complications and improve health outcomes [12,13]. Healthcare professionals are essential in tackling the burden of chronic diseases. However, the growing strain on healthcare system resources renders it necessary to secure alternative strategies to help manage population health [14].

Community pharmacies are accessible and convenient healthcare settings that are often the first point of contact for individuals seeking healthcare advice. Their long opening hours and geographical coverage allows pharmacies to reach a wide range of people [15]. Pharmacist intervention has proven effective in early detection of people at high risk of developing T2D [15,16].

Studies have shown that community pharmacy screening programs are effective in disease prevention and control [17,18]. As members of multidisciplinary teams, pharmacists can also help to improve therapeutic outcomes [19,20], reduce financial burdens [16], and play a key role in diabetes management [18,19]. Hence, these highly qualified and easily accessible health professionals are ideally placed to assist in implementing preventive health strategies, which are essential to prevent or delay the onset of diabetes and its complications [13].

From the outset of the COVID-19 pandemic, diabetes emerged as an indicator for higher rates of severe infection and mortality suggesting that diabetes was prevalent in patients hospitalized with the infection. Previously, studies have shown that patients with diabetes were more susceptible to Middle East Respiratory Syndrome (MERS) and Severe Acute Respiratory Syndrome (SARS) infection, due to dysregulated immune response leading to severe and extensive lung pathology [21,22].

Recent studies reported that newly diagnosed diabetes is commonly observed in COVID-19 patients, therefore raising concerns regarding a bi-directional relationship between the two health conditions. As the COVID-19 pandemic has progressed, there is growing evidence that after the acute phase of the disease, people with COVID-19 can develop lingering sequelae that may involve pulmonary and extrapulmonary organ system manifestations, such as diabetes [23,24].

The Finnish Diabetes Risk Score—FINDRISC is a validated, low-cost, simple, and non-invasive tool for assessing the risk of developing T2D within 10 years [25]. It was developed by the Finnish Diabetes Association and is based on a set of simple questions that assess lifestyle factors and medical history [26]. The tool consists of eight questions regarding age, body mass index, waist circumference, physical activity, fruit and vegetable consumption, history of high blood glucose, history of hypertension, and family history of diabetes. The score is calculated based on the responses to these questions, and individuals are categorized as very low, low, moderate, high, or very high risk of developing T2D in the following 10 years [27]. The tool has been shown to be effective in identifying individuals at high risk of developing T2D in different countries [17].

This study aims to report the results of post-pandemic pharmacy-based risk screening of T2D using the Finnish Diabetes Risk Score (FINDRISC) and compare the results using a another FINDRISC study in a comparable population before the COVID-19 pandemic.

## 2. Materials and Methods

### 2.1. Study Design and Participants

This study was a cross-sectional analytical study conducted in five community pharmacies in the central region of Portugal. Participants were recruited using convenience sampling from individuals who visited the pharmacy between the months of January and June 2023 and the months of January and May 2024. Eligible participants were adults aged 40 years and older, with no prior diagnosis of diabetes, who were able to provide informed consent.

### 2.2. Data Collection

Data collection was carried out by a trained pharmacist who administered the FINDRISC questionnaire, validated for use in Brazilian Portuguese [28], to eligible participants, using face-to face interviews. The pharmacist explained the purpose of the study and obtained informed consent from the participants before beginning. The questionnaire was administered in Portuguese, the native language of the participants. The FINDRISC score, ranging from 0 to 26, was calculated by adding the points of each response to the eight questions. The participants were then categorized as being very low risk (<7 points), low risk (7–11 points), moderate risk (12–14 points), high risk (15–20 points), and very high risk (>20 points).

Weight (in light clothing and without shoes) and waist circumference were collected using an Omron BF400 scale and a standard tape measure. Self-reported data were used to assess height. BMI was calculated using the formula BMI = Weight (kg)/[Height (m)]^2^.

A random capillary blood glucose (RCBG) test was performed on all willing participants, using a Contour Next blood glucose meter (Ascensia Diabetes Care). If the participants stated that they had fasted for at least 8 h, the RCBG test was classified as a fasting capillary blood glucose (FCBG) test. When the result was above 140 mg/dL or above 100 mg/dL when participants had fasted for at least 8 h, and the FINDRISC score was above 15, a glycated hemoglobin (HbA1c) test was performed using cobas b 101 system (Roche Diagnostics).

In addition to the FINDRISC questionnaire, smoking habits and educational levels were also assessed. The participants were categorized in accordance with the maximum attained degree, following the National Education System [29]. Seven categories were considered illiterate, first cycle (grades one to four), second cycle (grades five and six), third cycle (grades seven to nine), upper secondary education (grades 10, 11 and 12), bachelor’s degree, and master’s degree.

### 2.3. Data Analysis

Data were analyzed using Microsoft Excel. The results are presented with number-percentage tables. Descriptive statistics are presented as frequencies and percentages (%) for categorical variables. Chi-square tests were performed to determine any association between the FINDRISC score and its components, by identifying significant differences between the observed and expected frequencies. The analyses were carried out by removing the score contribution of the component under analysis (e.g., age) from the FINDRISC total score and testing the association between this FINDRISC modified score and each component. Spearman’s rank correlation test was used to analyze the association between the education level and the FINDRISC total score. Results were considered statistically significant at a *p* < 0.05.

### 2.4. Ethical Considerations

The study was conducted in accordance with the ethical principles of the Declaration of Helsinki and the International Conference on Harmonization Good Clinical Practice guidelines. Informed consent was obtained from all participants before data collection. Participants were assured of the confidentiality and anonymity of their data, and all data were kept securely and only accessible to the study team.

## 3. Results

A total of 494 participants were interviewed in the study: 290 (58.7%) women and 204 (41.3%) men. The average FINDRISC score was 12.3, with women scoring higher (12.8) than men (11.8). Overall, 25.5% (126) of the participants presented a high-risk score, and the percentage of women (27.9%) in this group was higher than that of men (22.1%). There were 21 (4.3%) participants in the very high-risk group, 14 (4.8%) women and 7 (3.4%) men. There was no statistically significant relationship between gender and the FINDRISC score (*p* > 0.05). Results are shown in Table 1.

More than half of our study sample (51.4%) was aged over 64 years or older, 441 (89.3%) had a Body Mass Index (BMI) over 25, 249 (50.4%) had a larger waist circumference (men > 102 cm; women > 88 cm), and 66.8% (330) did not exercise regularly. In addition, 90.9% (449) of the participants reported no previous history of high blood glucose levels, and 54.1% (267) had no family history of DM. Regarding antihypertensive drug treatment, 58.8% (120) of men reported receiving treatment compared with 42.4% (123) of women. Fruit consumption was predominantly higher within our sample. The FINDRISC results are described in Table 2.

Table 3 presents the FINDRISC score for each component. The data presented in Table 3 indicates a clear relationship between age and FINDRISC scores. Among participants aged less than 45 years, 44.2% were categorized as very low risk, while this proportion decreased significantly with age, with only 4.7% of those over 64 years in the very low-risk category. Conversely, the percentage of participants classified as high risk or very high risk increased with age, with the highest proportion of high-risk individuals (31.3%) found in the 55–64 age group and 27.6% in the group over 64 years of age. The difference in risk scores by age was statistically significant (*p* < 0.05), highlighting age as a major factor influencing diabetes risk.

Similarly, the analysis of BMI and FINDRISC scores shows that individuals with a BMI < 25 had the highest proportion of very low risk (45.3%) and none were in the very high-risk category. In contrast, participants with a BMI > 30 were predominantly in the high-risk category (50.4%), with 11.6% in the very high-risk category. The association between BMI and diabetes risk was statistically significant (*p* < 0.05), confirming BMI as a strong predictor of diabetes risk.

For waist circumference, the data for both men and women reveal a significant relationship with FINDRISC scores. Among women with a waist circumference > 88 cm, 38.0% were classified as moderate risk and 36.9% as high risk. Similarly, among men with a waist circumference > 102 cm, 37.1% were classified as moderate risk and 38.6% as high risk. The *p*-value (<0.05) suggests that larger waist circumference is significantly associated with higher diabetes risk in both genders.

Physical activity also had a notable impact on FINDRISC scores. Among participants who reported regular exercise, 18.9% were classified as very low risk, compared to only 13.4% in the high-risk category. In contrast, 31.5% of those who did not engage in regular physical activity were in the high-risk group. The association between lack of physical exercise and elevated diabetes risk was statistically significant (*p* < 0.05), highlighting the protective role of physical activity in diabetes prevention.

Although fruit and vegetable consumption did not show a statistically significant relationship with FINDRISC scores (*p* = 0.10), there was a trend suggesting that participants who consumed fruits and vegetables regularly were less likely to have high or very high-risk scores. However, a higher proportion of those who did not consume sufficient fruits and vegetables fell into the moderate and high-risk categories.

The relationship between FINDRISC scores and a history of antihypertensive drug (AHD) treatment was also significant (*p* < 0.05). Among participants with a history of AHD use, 33.7% were classified as high risk, compared to only 17.5% in those without a history of AHD use. This indicates a potential association between hypertension and increased diabetes risk, possibly due to the metabolic effects of hypertension.

A history of high blood glucose (BG) was strongly associated with higher FINDRISC scores. Among individuals with a history of high BG, 35.6% were classified as very high risk, and 40.0% as high risk. This association was statistically significant (*p* < 0.05), emphasizing the importance of monitoring blood glucose levels as a key indicator of diabetes risk. Finally, family history of diabetes, particularly in first-degree relatives, was significantly associated with higher FINDRISC scores (*p* < 0.05). Over 50% of participants with a first-degree relative with diabetes were classified as high risk, demonstrating the strong genetic predisposition to diabetes.

The data presented in Table 4 show a significant relationship between educational level and FINDRISC scores (*p* < 0.05), with lower educational levels associated with higher diabetes risk. A Spearman correlation test (rs = −0.214; *p* < 0.05) confirms that lower levels of education are associated with a higher risk of diabetes. Participants with first cycle education had the highest proportion of individuals in the moderate (39.9%), high (37.3%), and very high-risk (38.1%) categories, indicating that lower education correlates with increased diabetes risk. Similarly, participants with second and third cycle education also showed elevated risk, though a smaller percentage fell into the higher-risk categories compared to those with only first cycle education.

In contrast, individuals with upper secondary education exhibited a more balanced distribution across risk categories, with 24.5% in the very low-risk category and 23.0% in the high-risk group, suggesting that while risk decreases with higher education, it is still prevalent. Bachelor’s degree holders had a relatively lower diabetes risk, with 26.4% classified as very low risk and only 7.1% in the high-risk category, further underscoring the protective effect of higher education.

The lowest diabetes risk was observed among participants with a master’s degree, where a majority were in the very low or low-risk categories, and none were classified as very high risk. This trend indicates that higher educational levels are associated with a reduced risk of diabetes, likely reflecting better access to health knowledge and resources.

Table 5 examines the relationship between smoking habits and FINDRISC scores, comparing smokers and non-smokers across different diabetes risk categories. While the results show no statistically significant association between smoking and diabetes risk (*p* = 0.76), some trends can still be noted. Among smokers, 12.5% were categorized as very low risk (<7), with the majority (35.4%) falling into the low-risk category (7–11). A smaller percentage (27.1%) of smokers were classified as high risk (15–20), and only 2.1% were in the very high-risk category (>20).

In comparison, non-smokers had a similar distribution, with 10.5% in the very low-risk category and 30% in the low-risk group. However, a larger proportion of non-smokers (29.6%) were classified as moderate risk (12–14), while 25.3% were in the high-risk group (15–20). Notably, 4.5% of non-smokers were in the very high-risk category, slightly more than among smokers.

Table 6 presents the mean results of random capillary blood glucose (RCBG) levels and fasting capillary blood glucose (FCBG) levels according to FINDRISC score. The mean levels rise with the increase of the risk group, except for the FCBG mean result for the moderate risk group. One participant included in this group presented an FCBG level of 469 mg/dL. According to our methods, this participant was not eligible for an Hb1Ac test. However, given the result, an Hb1Ac test was administered, the result was 13,2%. The participant’s GP was immediately contacted.

**Figure 1:**
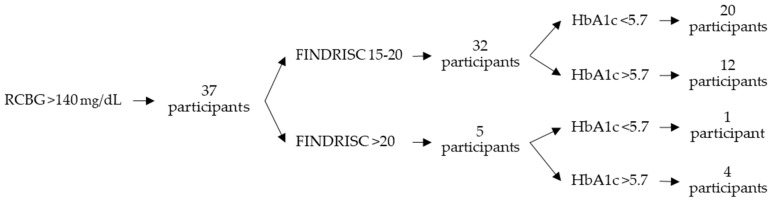
Distribution of HbA1c results by FINDRISC score of the participants with RCBG levels above 140 mg/dL.

**Figure 2:**
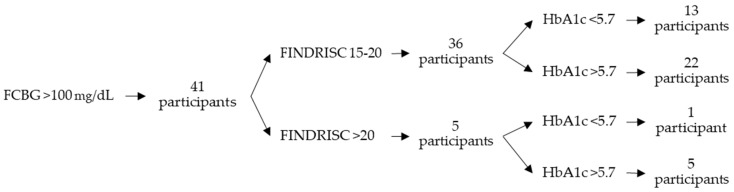
Distribution of HbA1c results by FINDRISC score of the participants with FCBG levels above 100 mg/dL.

Of the 494 participants, 78 included in the high or very high-risk group presented elevated capillary blood glucose levels. Of these, 37 were administered an RCBG test and presented levels above 140 mg/dL, and 41 were administered an FCBG test and presented levels above 100 mg/dL. An Hb1Ac test was administered to these 78 participants, and 43 (55.1%) presented results above 5.7%. Of these, 34 were included in the high-risk group (27.0% of the participants included in the high-risk group) and 9 were included in the very-high-risk group (42.9% of the participants included in the very-high-risk group). Table 7 shows the results by FINDRISC score. Figure 1 shows the distribution of HbA1c results by FINDRISC score of the participants with RCBG levels above 140 mg/dL and Figure 2 shows those with FCBG levels above 100 mg/dL.

As the FINDRISC score increases, the percentage of participants with elevated capillary blood glucose levels rises. Also, the percentage of participants in the very-high-risk group with Hb1Ac above 5.7% is higher when compared to those with Hb1Ac below 5.7%. This may provide some validation to the FINDRISC test.

All participants included in the high and very high-risk groups were informed of the risks of T2D. The advantages of healthy eating and regular exercise were thoroughly discussed, and the participants were advised to monitor their blood glucose levels more frequently. Participants with Hb1Ac above 5.7% were referred to a general practitioner (GP).

## 4. Discussion

This study found that Portuguese community pharmacies can identify patients at risk of developing T2D by using easy and cost-effective screening tools. Our study found that 29.8% (147) of the participants were included in the high or very high-risk group and that 8.7% (43) were eligible for referral to a GP, results similar to other studies [15,30].

Community pharmacies can play an important role in informing patients of the benefits of exercising regularly and can also give advice regarding balanced diets. Their proximity to the community puts them in an excellent position to monitor patients and help them reduce major modifiable risk factors like obesity [31] and sedentary behavior. As mentioned, all participants in our study included in the high and very-high risk groups were given advice regarding healthy eating habits and the benefits of regular exercise. Also, they were encouraged to return to the pharmacy regularly to monitor their weight and blood glucose levels.

Of all the parameters evaluated in the questionnaire, only the ingestion of fruits/vegetables showed no statistically significant relationship with the risk of developing D2T in ten years. The latter complies with the results of the original FINDRISC study in which the daily consumption of vegetables, fruits, and berries was not statistically significant; however, this parameter was kept in the model to emphasize the importance of diet in the prevention of diabetes [32].

The lack of regular physical activity can increase the risk of T2D [33,34]. Also, the risk of developing T2D has been associated with BMI [35,36]. In our study, 66.8% (330) of the participants reported not exercising regularly, and the percentage of women (41.5%) in this group was higher than that of men (25.3%). Moreover, the percentage of participants with BMI above 30 was 89.3%.

Our analysis, similar to the one by Mavrogianni et al., demonstrates significant associations between FINDRISC scores and various demographic, lifestyle, and clinical factors. Age, BMI, waist circumference, lack of exercise, history of high BG, and family history of diabetes are all strongly correlated with increased diabetes risk. Furthermore, regular physical exercise and maintaining a lower BMI or waist circumference were key protective factors against developing diabetes [37].

Although smoking does not appear to be a major factor in diabetes risk based on these findings, the data suggests that smokers may have a slightly higher tendency to fall into the high-risk category, while non-smokers show a greater distribution in the moderate and very high-risk categories [38]. Overall, in our study smoking habits alone do not seem to significantly influence FINDRISC scores compared to other factors such as age, BMI, and education.

One of the objectives of this analysis was to compare if the COVID-19 pandemic had any effect on the FINDRISC scores within similar populations. Several authors suggest a potential relationship between SARS-CoV-2 infection and the development of new-onset diabetes. The proposed mechanism indicates that the virus enters cells by attaching to angiotensin-converting enzyme 2 (ACE-2) receptors found on various organs and tissues. Once inside, the virus releases its RNA, which the cell uses to replicate, producing viral proteins and RNA. These products are assembled and released from the cell to infect others. During this process, ACE-2 downregulation impairs the conversion of angiotensin II to angiotensin [1,2,3,4,5,6,7], which leads to a buildup of angiotensin II. This accumulation causes problems like inflammation, lung damage, and blood clots, and it may contribute to the development of new-onset diabetes in COVID-19 patients [24,39].

The results from our work, when compared to the results obtained in a Portuguese pre-pandemic FINDRISC study by Murteira et al., show similar results regarding each variable and its correspondent FINDRISC score. Both studies showed statistically significant relationships for age, BMC, waist circumference, physical activity, AHD, high BG, and family history of diabetes. Similarly, both studies matched the non-statistically significant relationships in the gender and fruit or vegetable consumption variables. Compared to our work, this analysis did not include smoking habits and educational levels [17].

Furthermore, another pre-pandemic FINDRISC study by Milovanovic et al. performed in Spain and Italy showed a match of statistically significant relationships in age, BMC, waist circumference, physical activity, AHD, high BG, family history of diabetes, and education level. Similarly, both studies matched the non-statistically significant relationships concerning gender. Our work reveals a difference regarding smoking habits and fruit or vegetable consumption variables, both with non-statistically significant relationships as opposed to the results obtained in the pre-pandemic study [40].

Our study shows that the COVID-19 pandemic did not have a major impact on the FINDRISC screening tool. The studies used in our comparison measured all the variables used in ours and did not show significant changes in variables like high blood glucose, BMC, physical activity, waist circumference, and antihypertensive medication, which could be more related to developing diabetes. Fruit consumption and smoking did show differences in one of the pre-pandemic studies compared to ours, which might suggest a change in some personal health habits. Additionally, the prevalence of COVID-19 cases, as per the monthly reports of the Portuguese General Directorate of Health, show that during the period of this study, there was a total of 39,114 cases between January and June 2023 and 6101 cases between January and May 2024. Since these reports show the number of cases on a national scale, we were not able to measure this prevalence in the region of our study. However, it is possible to see a decrease in the number of cases, which could be explained due to the effective vaccination plans and infection contention measures all throughout the country [41].

This decrease was due to the strict quarantine implemented all throughout Portugal during the pandemic, thus limiting the contact between the population and their movement within the country. Another issue regarding the restrictions during the pandemic was the lifestyle modifications, such as eating habits and exercise (assessed in the FINDRISC score). Even though we were not able to measure if any eating habits were changed, it is possible that exercise habits changed due to the movement restrictions imposed during the pandemic such as the closing of gyms and other exercise facilities.

Health literacy is the ability to obtain, understand, and use information to improve health status [42]. Studies have shown that it is related to educational levels [43] and plays a major role in health management [41]. Regarding educational level and FINDRISC scores, our study suggests that participants with lower educational levels, particularly those with first cycle education or lower, tended to have higher diabetes risk scores, with a large proportion falling into the moderate, high, and very high-risk categories. In contrast, participants with higher educational attainment, such as those with a bachelor’s or master’s degree, were more likely to be in the very low or low-risk categories. This trend suggests that higher educational levels may be associated with a lower risk of developing diabetes.

Healthcare professionals can help raise individual and community health literacy [43]. They can inform patients of the benefits of adopting healthy lifestyles and help them understand why this is so important. Community pharmacists are highly qualified health professionals, trusted by patients and easily available to clarify any questions they may have pertaining to their health. Also, when needed, they can refer patients to the GP or to another health professional (i.e., psychologist, nutritionist). In our study, 43 patients, identified as high or very-high risk and with Hb1Ac test above 5.7%, were referred to the GP.

Finally, to prevent individuals with moderate or lower FINDRISC scores from progressing to higher risk levels, the role of community pharmacists will be crucial. Through regular blood glucose screenings and targeted public health initiatives, such as promoting lifestyle changes, healthy eating habits, and active health monitoring, pharmacists can help prevent diabetes. These efforts will also enhance patients’ awareness of potential health complications, empowering them to take proactive measures in managing their health [17].

### Limitations

There are some limitations to this study. The study was conducted in five community pharmacies in Portugal, which may limit the generalization of the findings. It used a convenience sampling, which may introduce selection bias. The FINDRISC questionnaire relies on self-reported data, which may be subject to recall bias or social desirability bias.

## 5. Conclusions

Community pharmacies can play an important role in screening for diabetes using easy, cost-effective validated tools such as FINDRISC. Screening for diabetes in community pharmacies can help to identify individuals at risk of developing diabetes who may not otherwise seek medical attention, lead to early detection and intervention, and improve the overall health of the community.

The study highlighted several modifiable risk factors, such as lack of physical activity and high BMI, particularly among women. These findings emphasize the importance of community pharmacies in educating patients about a healthy lifestyle, such as regular exercise and balanced diets, and encouraging them to return for monitoring. Additionally, the significant relationship between lower educational levels and increased T2D risk calls attention to the need for improving health literacy. As accessible and trusted health professionals, pharmacists are ideally placed to enhance community health by providing personalized advice, supporting health literacy, and referring high-risk patients to other healthcare providers. This reinforces the vital role pharmacies can play in reducing T2D risk and promoting better overall health in the community.

Finally, our findings suggest that the COVID-19 pandemic did not significantly alter the overall risk profile for developing type 2 diabetes (T2D), as assessed by the FINDRISC tool. The relationship between key risk factors, such as age, body mass index, waist circumference, physical activity, and family history of diabetes, remained consistent with pre-pandemic studies. These findings imply that the established predictors of T2D, such as obesity and sedentary behavior, continue to play a dominant role in diabetes risk, and the pandemic has not drastically altered this landscape. However, it remains crucial to monitor the long-term impact of COVID-19 on metabolic health and chronic disease risk in future studies.

## Figures and Tables

**Table 1:** Distribution of the FINDRISC scores, as reported on the questionnaires.

FINDRISC	Men	Women	Total	*p*
	*n* = 204	*n* = 290	*n* = 494	
Score	*n* (%)	*n* (%)	*n* (%)	
<7	28 (13.7)	25 (8.6)	53 (10.7)	
>7 < 11	71 (34.8)	80 (27.6)	151 (30.6)	
>12 < 14	53 (26.0)	90 (31.1)	143 (29.0)	>0.05
>15 < 20	45 (22.1)	81 (27.9)	126 (25.5)	
>20	7 (3.4)	14 (4.8)	21 (4.3)	
**Mean**	11.8	12.8	12.3	

**Table 2:** Sample characteristics and results of the FINDRISC questionnaire.

FINDRISC	Score	Men		Women		Total	
		(*n* = 204)		(*n* = 290)		(*n* = 494)	
		*n* (%)		*n* (%)		*n* (%)	
**Age (yr)**							
<45	0	18	(8.8)	34	(11.7)	52	(10.5)
45–54	2	33	(16.2)	56	(19.3)	89	(18.0)
55–64	3	33	(16.2)	66	(22.8)	99	(20.1)
>64	4	120	(58.8)	134	(46.2)	254	(51.4)
**BMI (kg/m^2^)**							
<25	0	15	(7.4)	38	(13.1)	53	(10.7)
25–30	1	144	(70.6)	176	(60.7)	320	(64.8)
>30	3	45	(22.1)	76	(26.2)	121	(24.5)
**Waist circumference (cm)**							
men < 94; women < 80	0	78	(38.2)	32	(11.0)	110	(22.3)
men 94–102; women 80–88	3	56	(27.5)	79	(27.3)	135	(27.3)
men > 102; women > 88	4	70	(31.3)	179	(61.7)	249	(50.4)
**Physical exercise > 30 min/day**							
no	2	125	(61.3)	205	(70.7)	330	(66.8)
yes	0	79	(38.7)	85	(29.3)	164	(33.2)
**Fruit/vegetables**							
no	1	66	(32.4)	64	(22.1)	130	(26.3)
yes	0	138	(67.6)	226	(77.9)	364	(73.7)
**History AHD treatment**							
no	0	84	(41.2)	167	(57.6)	251	(50.8)
yes	2	120	(58.8)	123	(42.4)	243	(49.2)
**History high BG**							
no	0	194	(95.1)	255	(87.9)	449	(90.9)
yes	5	10	(4.9)	35	(12.1)	45	(9.1)
**Family history of diabetes**							
no	0	113	(55.4)	154	(53.1)	267	(54.1)
second degree	3	31	(15.2)	36	(12.4)	67	(13.6)
first degree	5	60	(29.4)	100	(34.5)	160	(32.4)

Legend: FINDRISC—Finnish Diabetes Risk Score; BMI—body mass index; AHD—antihypertensive drug; BG—blood glucose.

**Table 3:** FINDRISC scores for each component.

FINDRISC Score	Age (yr) *n* = 494				
	<45	45–54	55–64	>64	*p*
	*n* (%)	*n* (%)	*n* (%)	*n* (%)	
**Very low (<7)**	23 (44.2)	8 (9.0)	10 (10.1)	12 (4.7)	
**Low (7–11)**	17 (32.7)	35 (39.3)	24 (24.2)	75 (29.5)	
**Moderate (12–14)**	8 (15.4)	23 (25.8)	29 (29.3)	83 (32.7)	<0.05
**High (15–20)**	4 (7.7)	21 (23.6)	31 (31.3)	70 (27.6)	
**Very high (>20)**	0 (0.0)	2 (2.2)	5 (5.1)	14 (5.5)	
**Total**	52 (100.0)	89 (100.0)	99 (100.0)	254 (100.0)	
	**BMI *n* = 494**				
	**<25**	**25–30**	**>30**		***p***
	***n* (%)**	***n* (%)**	***n* (%)**		
**Very low (<7)**	24 (45.3)	29 (9.1)	0 (0.0)		
**Low (7–11)**	15 (28.3)	127 (39.7)	9 (7.4)		
**Moderate (12–14)**	8 (15.1)	98 (30.6)	37 (30.6)		<0.05
**High (15–20)**	6 (3.1)	59 (18.4)	61 (50.4)		
**Very high (>20)**	0 (0.0)	7 (2.2)	14 (11.6)		
**Total**	53 (100.0)	320 (100.0)	121 (100.0)		
	**Waist circumference/Women (cm) *n* = 290**				
	**<80**	**80–88**	**>88**		***p***
	***n* (%)**	***n* (%)**	***n* (%)**		
**Very low (<7)**	18 (56.3)	7 (8.9)	0 (0.0)		
**Low (7–11)**	8 (25.0)	40 (50.6)	32 (17.9)		
**Moderate (12–14)**	5 (15.6)	17 (21.5)	68 (38.0)		<0.05
**High (15–20)**	1 (3.1)	14 (17.7)	66 (36.9)		
**Very high (>20)**	0 (0.0)	1 (1.3)	13 (7.3)		
**Total**	32 (100.0)	79 (100.00)	179 (100.0)		
	**Waist circumference/Men (cm) *n* = 204**				
	**<94**	**94–102**	**>102**		***p***
	***n* (%)**	***n* (%)**	***n* (%)**		
**Very low (<7)**	25 (32.1)	3 (5.4)	0 (0.0)		
**Low (7–11)**	38 (48.7)	22 (39.3)	11 (15.7)		
**Moderate (12–14)**	13 (16.7)	14 (25.0)	26 (37.1)		<0.05
**High (15–20)**	2 (2.6)	16 (28.6)	27 (38.6)		
**Very high (>20)**	0 (0.0)	1 (1.8)	6 (8.6)		
**Total**	78 (100.0)	56 (100.0)	70 (100.0)		
	**Physical exercise *n* = 494**				
	**yes**		**no**		***p***
	***n* (%)**		***n* (%)**		
**Very low (<7)**	31 (18.9)		22 (6.7)		
**Low (7–11)**	59 (36.0)		92 (27.9)		
**Moderate (12–14)**	46 (28.0)		97 (29.4)		<0.05
**High (15–20)**	22 (13.4)		104 (31.5)		
**Very high (>20)**	6 (3.7)		15 (4.5)		
**Total**	164 (100.0)		330 (100.0)		
	**Fruit/Vegetables *n* = 494**				
	**yes**		**no**		***p***
	***n* (%)**		***n* (%)**		
**Very low (<7)**	40 (11.0)		13 (10.0)		
**Low (7–11)**	123 (33.8)		28 (21.5)		
**Moderate (12–14)**	99 (27.2)		44 (33.8)		0.10
**High (15–20)**	88 (24.2)		38 (29.2)		
**Very high (>20)**	14 (3.8)		7 (5.4)		
**Total**	364 (100.0)		130 (100.0)		
	**History of AHD treatment *n* = 494**				
	**yes**		**no**		***p***
	***n* (%)**		***n* (%)**		
**Very low (<7)**	5 (2.1)		48 (19.1)		
**Low (7–11)**	58 (23.9)		93 (37.1)		
**Moderate (12–14)**	83 (34.2)		60 (23.9)		<0.05
**High (15–20)**	82 (33.7)		44 (17.5)		
**Very high (>20)**	15 (6.2)		6 (2.4)		
**Total**	243 (100.0)		251 (100.0)		
	**History of high BG *n* = 494**				
	**yes**		**no**		***p***
	***n* (%)**		***n* (%)**		
**Very low (<7)**	0 (0.0)		53 (11.8)		
**Low (7–11)**	3 (6.7)		148 (33.0)		
**Moderate (12–14)**	8 (17.8)		135 (30.1)		<0.05
**High (15–20)**	18 (40.0)		108 (24.1)		
**Very high (>20)**	16 (35.6)		5 (1.1)		
**Total**	45 (100.0)		449 (100.0)		
	**Family history of diabetes *n* = 494**				
	**no**	**2nd degree**	**1st degree**		***p***
	***n* (%)**	***n* (%)**	***n* (%)**		
**Very low (<7)**	43 (16.1)	9 (13.4)	1 (0.6)		
**Low (7–11)**	114 (42.7)	21 (31.3)	16 (10.0)		
**Moderate (12–14)**	72 (30.7)	20 (29.9)	41 (25.6)		<0.05
**High (15–20)**	27 (10.1)	16 (23.9)	83 (51.9)		
**Very high (>20)**	1 (0.4)	1 (1.5)	19 (11.9)		
**Total**	267 (100.0)	67 (100.0)	160 (100.0)		

Legend: FINDRISC—Finnish Diabetes Risk Scores; BMI—body mass index; AHD—antihypertensive drug; BG—blood glucose.

**Table 4:** FINDRISC scores according to educational level.

	FINDRISC Score					
	<7	7–11	12–14	15–20	>20	*p*
	*n* (%)	*n* (%)	*n* (%)	*n* (%)	*n* (%)	
Illiterate	0 (0.0)	0 (0.0)	1 (0.7)	1 (0.8)	1 (4.8)	
First cycle	10 (18.9)	42 (27.8)	57 (39.9)	47 (37.3)	8 (38.1)	
Second cycle	1 (1.9)	23 (15.2)	14 (9.8)	12 (9.5)	1 (4.8)	
Third cycle	10 (18.9)	25 (16.6)	19 (13.3)	23 (18.3)	4 (19.0)	<0.05
Upper secondary education	13 (24.5)	33 (21.9)	30 (21.0)	29 (23.0)	4 (19.0)	
Bachelor’s degree	14 (26.4)	22 (14.6)	13 (9.1)	9 (7.1)	3 (14.3)	
Master’s degree	5 (9.4)	5 (3.3)	5 (3.5)	4 (3.2)	0 (0.0)	
NA	0 (0.0)	1 (0.7)	4 (2.8)	1 (0.8)	0 (0.0)	

Legend: NA—information not provided.

**Table 5:** FINDRISC scores according to smoking habits.

FINDRISC Score	Smoker *n* = 494		*p*
	Yes	No	
	*n* (%)	*n* (%)	
**Very low (<7)**	6 (12.5)	47 (10.5)	
**Low (7–11)**	17 (35.4)	134 (30.0)	
**Moderate (12–14)**	11 (22.9)	132 (29.6)	0.76
**High (15–20)**	13 (27.1)	113 (25.3)	
**Very high (>20)**	1 (2.1)	20 (4.5)	
**Total**	48 (100.0)	446 (100.0)	

**Table 6:** Mean of random and fasting capillary blood glucose levels according to FINDRISC score.

FINDRISC	Mean	Mean
Score	RCBG mg/dL	FCBG mg/dL
<7	118	98
>7 < 11	121	100
>12 < 14	123	122
>15 < 20	127	112
>20	146	112

Legend: RCBG—random capillary blood glucose; FCBG—fasting capillary blood glucose.

**Table 7:** Participants with elevated blood glucose levels and glycated hemoglobin, categorized by high and very-high FINDRISC scores.

	FINDRISC Score				
	<7	7–11	12–14	15–20	>20
	(*n* = 53)	(*n* = 151)	(*n* = 143)	(*n* = 126)	(*n* = 21)
	*n* (%)	*n* (%)	*n* (%)	*n* (%)	*n* (%)
RCBG > 140 mg/dL	8 (15.1)	16 (10.6)	20 (14.0)	32 (25.4)	5 (23.8)
FCBG > 100 mg/dL	2 (3.8)	11 (7.3)	11 (7.7)	35 (27.8)	6 (28.6)
HbA1c < 5.7%	-	-	-	33 (26.2)	2 (9.5)
HbA1c > 5.7%	-	-	-	34 (27.0)	9 (42.9)

Legend: RCBG—random capillary blood glucose; FCBG—fasting capillary blood glucose; HbA1c—glycated hemoglobin.

## Data Availability

The datasets generated and/or analyzed during the current study are not publicly available due to protecting the privacy of study participants, according to the Portuguese General Data Protection Regulation, but are available from the corresponding author on reasonable request.
